# Prevalence and factors associated to gestational diabetes mellitus among pregnant women in Libreville: a cross-sectional study

**DOI:** 10.11604/pamj.2022.41.129.28710

**Published:** 2022-02-15

**Authors:** Elisabeth Lendoye, Edgard Brice Ngoungou, Opheelia Makoyo Komba, Benjamin Ollomo, Marie-Andrée N'negue-Mezui, Serge Bekale, Lauriane Yacka-Mouele, Brice Wilfried Obiang Obounou, Marie-Pierrette Ntyonga-Pono, Edouard Ngou-Milama

**Affiliations:** 1 Department of Chemistry and Biochemistry, Faculty of Medicine, University of the Health Sciences, Libreville, Gabon,; 2 Biostatistics and Medical Informatics (DEBIM), Faculty of Medicine, University of the Health Sciences, Libreville, Gabon,; 3 Research Unit in Epidemiology of Chronic Diseases and Health Environment (UREMCSE), University of the Health Sciences, Libreville, Gabon,; 4 Department of Gynecology and Obstetrics, University of the Health Sciences, Libreville, Gabon,; 5 Department of Gynecology and Obstetrics Service, Libreville University Hospital Centre, Libreville, Gabon,; 6 Department of Biochemistry, Joint Research Units from Biomed Cirmf, Faculty of Medicine, Libreville, Gabon,; 7 Department of Food Nutrition, College of Natural Sciences, Keimyung University Dalgubeoldaero, Dalseo-gu, Daegu, Korea,; 8 Department of Endocrinology, Faculty of Medicine, University of the Health Sciences, Libreville, Gabon,; 9 Endocrinology Service, Libreville University Hospital Centre, Libreville, Gabon

**Keywords:** Gestational diabetes mellitus, prevalence, oral glucose tolerance test, Gabon, Africa

## Abstract

**Introduction:**

mainly occurring in low and middle income countries, gestational diabetes mellitus (GDM) represents 84% of hyperglycemia during pregnancy throughout the world. Moreover, being black is a risk factor to develop the disease. Our objective was to determine the prevalence and the associated factors of GDM in Libreville (Gabon).

**Methods:**

a cross-sectional study was carried out. Known diabetic women were excluded from the study and we had submitted asymptomatic pregnant women to a 2 steps 75g oral glucose tolerance test (T0-T2H), regardless of the stage of pregnancy at the moment of recruitment. The threshold for positivity was set at blood glucose level ≥ 8.5mmol/L World Health Organization (WHO 2013 threshold) and ≥ 7.8mmol/L (WHO 1999 threshold). Data were analyzed using Statview® for descriptive statistics, for both bivariate and multivariate analysis.

**Results:**

among 245 participants, we have found a GDM prevalence of 10.2% according to WHO 1999 threshold and 4.5% according to WHO 2013 threshold. Applying the WHO 1999 threshold, the associated factors were high maternal weight (p= 0.0498), overweight at recruitment (p=0.0246), personal history of GDM (p< 0.0001), age becomes an associated factor only if it is combined with high parity (p=0.0061). ceaserian-section and macrosomia were the two outcomes of GDM.

**Conclusion:**

Libreville has a high prevalence of GDM when the WHO 1999 criteria is compared to the WHO 2013 criteria. Discordance is also found with the identified associated factors. Further studies are needed to better appreciate gestational diabetes in Gabon.

## Introduction

Infectious diseases, compared to non-communicable diseases (NCDs), remain a major public health´s issue in sub-Saharan Africa [1, 2]. There are around 20 million live births each year in the world, of which 1 out of 6 among them is associated with hyperglycemia during pregnancy, with gestational diabetes mellitus (GDM) accounting for 84% [3]. The majority of hyperglycemia during pregnancy occurs in low and middle-income countries, where access to maternal care may be limited and consequently responsible for a higher incidence of both maternal and fetal morbidity and mortality [3]. It is well established that GDM has a negative impact on the health of both the mother and the child to be born, resulting in a high incidence of both fetal and maternal morbidity [3]. Maternal mortality is still high in Gabon, with 271 deaths per 100,000 live births [4]. This figure places Gabon well above the global average for developing countries, where the maternal mortality ratio in 2015 was 239 deaths per 100,000 live births, compared to 12 per 100,000 in developed countries [5]. The most common maternal complication of GDM is C-section [6, 7]. For newborns, the main complication is macrosomia and its traumatic consequences [6, 7]. In the long run, mother and baby remain at increased risk of developing type 2 diabetes (T2D) in about 50% of cases [6]. In sub-Saharan Africa, the prevalence of GDM varies between 1% and 13.9% [8].

A more recent meta-analysis that had pooled data from 33 studies estimated an overall prevalence of gestational diabetes in sub-Saharan Africa of 9% (95%CI, 7-12%) [9]. These prevalence have only been assessed in 6 countries of this region of the world [8, 9]. Furthermore, little information has been published on the risk factors of GDM in black sub-Saharan African populations. Therefore, classic risk factors such as the previous history of gestational diabetes, age, overweight, etc. are “de facto” considered as risk factors of GDM in sub-Saharan Africa [8, 10]. Thus, this disease would affect more people of African origin and the pre-existing overweight would be more involved in the occurrence of GDM than excess weight acquired during pregnancy [10, 11]. In another study previously carried out by our team, we had shown that fetuses were more insulin-sensitive than their mothers (adiponectin levels three times higher in cord blood than in mothers) near the end of the pregnancy [12]. It was also found that fetal macrosomia results in the case of more pronounced mother's glucose intolerance, such as GDM. It is recognized that an African ethnic origin is a risk factor for developing GDM as well as being overweight or obese [10, 11]. In Gabon, more than half of Gabonese women (57.1%) are overweight or obese [4]. Consequently, due to the lack of data on the prevalence of GDM in Gabon, it seemed appropriate to carry out this study with the objective to evaluate the prevalence and factors associated with GDM in Libreville.

## Methods

**Study design:** we have selected 9 maternal and child health centers in Libreville and its suburbs in order to have a better representation of the population living in and around Libreville. Pregnant women were enrolled at each center (Table 1) where we had stayed for a week. These health centers were chosen because they offer the largest follow-up of insured pregnant women; all pregnant Gabonese women are registered to the insurance fund of health and social guarantee (in short and in French, also named *CNAMGS*) since January 2011. With the implementation of the health insurance system in Gabon, pregnancy follow-up is 100% free of charge in these public health centers [13]. Finally, another factor of selection is accessibility: these health structures are most accessible to the population, especially those in working-class neighborhoods, taking into account for geographical and financial factors. Although our study was not an exhaustive survey of the population, it is nevertheless a correct reflection of the follow-up of pregnant women without known pathologies in Libreville. We also chose Libreville because 89.37% of the Gabonese population resides in urban areas and more than 50% in Libreville and its suburbs [14].

**Table 1:** centers, in charge of maternal and child health, were study have been realized

Name of the center	Number of pregnant women identified in each center	Geographic localization	%
Awendje	40	Libreville downtown (166)	67.75
Egyptian-Gabonese Hospital	35		
Glass	23		
La peyrie	26		
Louis	25		
Private clinic	17		
Melen Hospital	51	East suburb	20.81
Owendo	7	South suburb	2.86
Okala	21	North suburb	8.58

**Study period:** we carried out a descriptive and analytical cross-sectional study from October 1^st^, 2014, to April 30^th^, 2015 in Libreville and its suburbs.

### Study population

**Selection criteria:** asymptomatic pregnant women that had given their written and informed consent and have been living in Gabon for at least two years were included in this study. There is no consensus in the scientific community for a recommended threshold with respect to the diagnosis of gestational diabetes. Two threshold values are currently used in this sense. One is the WHO 1999 threshold [15] and the other is the WHO 2013 threshold [16]. To diagnose GDM, we have opted for these two threshold values and we also took into account the criteria of the International Association of Diabetes and Pregnancy Study Groups (IADPSG) [17] which are based the following abnormal blood glucose values: fasting, 1 hour or 2 hours of 75g oral glucose tolerance test (OGTT) which allow the GDM to be diagnosed at any time during pregnancy [17]. Finally, due to the lack of consensus on diagnostic threshold values, we have decided to perform a 2-step 75g OGTT to pregnant women (T0 and T2h), regardless of the age of pregnancy at the time of enrollment with a blood glucose level ≥ 8.5mmol/L (WHO 2013 threshold) [16] and ≥ 7.8mmol/L (WHO 1999 threshold) [15] threshold for positivity.

**Exclusion criteria:** diabetic women or women who didn´t give us an informed and written consent were excluded from this study.

**Sampling:** the sample size was calculated using OpenEpi, version 3, SSPropor [18]. Due to the the lack of data on GDM prevalence in Gabon, we used results obtained in 2013 in Cameroon (6.3%) [19], a neighboring country with a similar GDM prevalence rate. There is a 5% margin of error (α), a power of 80% with a precision of 3.1%; the studied sample size was 236.

**Data collection:** the purpose and procedures of the study were discussed with pregnant women each day before enrollment began. Data was collected using a standardized data collection form. All pregnant women included in this study were subsequently contacted after their expected delivery date in order to record the anthropometric data of their respective newborns and their delivery modalities. For each participant, we took blood samples of peripheral venous blood in sodium fluoride and potassium oxalate tubes (5ml) for glucose determination. Once collected, blood samples were stored in a cooler at 4°C and immediately sent to the clinical biochemistry laboratory of the faculty of medicine. Plasma glucose levels were determined daily in each sample using enzyme tests with the Mindray BS-200® chemistry analyzer (Shenzhen, China).

### Studied variables

**Pregnant women:** we have collected socio-demographic data, parity, socio-economic level (occupation and living area), personal history of GDM (PHD) or familial history of T2D (FHD), delivery mode, gestational age at delivery and gestational age at the OGTT. Gestational age was first determined by the last menstrual period date and confirmed by ultrasonographic evaluation. This last data was collected from the pregnancy health record of each pregnant woman.

**Newborns:** we recorded their weights with respect to their mother´s gestational age at delivery. Fetal macrosomia is defined as a birth weight greater than the 90^th^percentile of a reference curve of a given population, which corresponds to the average weight within the 10 highest values found in a reference population. For this purpose, the classification of newborns into “normal”, “low-birth weight: LBW”, or “macrosomes” was based on the academic thesis of Mengue C (Table 2) [20].

**Table 2:** weight scale of normal newborns in Libreville

Gestational age at delivery (weeks of gestation)	Low-birth weight's newborns according to weeks of gestation	High-birth weight's newborns (macrosomes) according to weeks of gestation
34	< 2159	
37	< 2350	> 3374
38	< 2413	> 3450
39	< 2477	> 3500
40	< 2540	> 3600
41	< 2960	> 3700
42	< 2667	> 3790
43	< 2731	> 3880
45		> 4060
Mean weight	2537 ± 248	3669 ± 233

**Statistical analysis:** descriptive statistics were used to summarize patients´ sociodemographic and clinical characteristics. Data was analyzed by using Statview 5.0 software (SAS Institute, Inc., Cary, NC, USA). The results from quantitative variables were presented as mean ± standard deviation and those from qualitative variables are expressed as percentages. For the categorical variables, including our dependent variable (blood glucose after 2h of 75-g OGTT, according to WHO 1999 and WHO 2013 criteria), we used the Pearson chi square test when the theoretical number was greater than or equal to 5. For theoretical numbers less than 5, we chose Fisher's exact test. For quantitative variables (numbers greater than 30), we chose the student's T test. For the values below 30 we had chosen the Mann-Whitney or Kruskal-Wallis tests. The significance level chosen for all the statistical analyses was 0.05.

**Ethical consideration:** this study was conducted with the approval of the Gabonese National Research Ethics Committee (N°003/2014/SG/P), the Gabonese Ministry of Health (N°0072/MS/SG/DGS), and the Gabonese Ministry of Scientific Research (N°00129/MERS/SG/DGRSI/DRS). All participants provided us with their signed and informed consent before participating in the study. The survey was anonymous and the confidentiality of the sdata was respected.

## Results

**Description of the studied population at enrollment:** Table 3 summarizes the characteristics of the 245 pregnant women enrolled in this study. The mean age was 27.2 ± 6.0 years (range 15 and 43 years). Their mean weight at enrollment was 71.6kg ± 15.3 (extremes 48 and 133kg), while the mean weight before pregnancy was 63.1kg ± 13.9. The mean body mass index (BMI) was 27.4 ± 5.6 compared to 24.2 ± 5.7 before pregnancy. The mean parity was 1.7 ± 1.9. Approximatively 29% of the studied population were primiparous, i.e. women who had never had a child (n= 70/245), 62% (n= 152/245) were pauciparous (women who had 1-4 births), and only 9.4% (n= 23/245) of pregnant women were multiparous (5 or more births). At last, the mean gestational age at enrollment was 29.7 ± 5.2 weeks.

**Table 3:** characteristics of the study population at recruitment

Characteristics	Number (n=245)	Percentage (%)
**Recruitment areas**		
Libreville	166	67.8
Suburbs	79	32.2
**Pregnant women's residence areas**		
Libreville	150	61.2
East suburb	41	16.7
South suburb	32	13.1
North suburb	22	9.0
**Age (years)**		
(15-25)	89	36.3
(25-35)	125	51.0
(35-and more)	31	12.7
**Pre-existing BMI (Kg/m^2^)**		
≤ 25	163	66.5
> 25	82	33.5
**BMI at recruitment (Kg/m^2^)**		
≤ 25	97	39.6
> 25	148	60.4
**Gestational age at recruitment (weeks of gestation)**		
< 24	23	9.4
24-28	88	35.9
> 28	134	54.7
**Parity**		
Primiparous (0)	70	28.6
Pauciparous (1-4)	152	62.0
Multiparous (≥ 5)	23	9.4
**Personal history of GDM**		
Yes	2	0.8
No	243
Yes	48	19.6
No	197	80.4

BMI: body mass index; GDM: gestational diabetes mellitus; T2D: type 2 diabetes.

**The prevalence of gestational diabetes mellitus:** after 75g OGTT, we identified 25 GDM, i.e. a prevalence of 10.2% applying WHO 1999 criteria (T2H ≥ 7.8 mmol/L). However, the prevalence was reduced to 4.5% based on WHO 2013 criteria (T2H ≥ 8.5 mmol/L).

### Description of the studied population at delivery

**Description of the pregnant women:** among the 25 women diagnosed with a GDM based on WHO 1999 criteria, we have recorded only one (4.2%) pre-term birth (before 37 weeks), 13 (54.2%) delivery between 37 and 40 weeks and, 10 (41.7%) over-term deliveries. Based on WHO 2013 criteria, we did not notice any pre-term birth, 63.6% of them delivered between 37 and 40 weeks and 36.4% over-term births. Only one case of co-infection with HIV and Syphilis was found in the study cohort (this fact was recorded among pregnant woman with GDM) and resulted in the birth of the only LBW newborn in the cohort when considering WHO 2013 criteria.

**Description of the newborns:** due to many 'drop-out', we were only able to compile data at delivery on half of the original sample (n=114/245). Most of these children were born by vaginal route (91.3%). There was a predominance of males with 67 boys against 60 girls, giving a sex ratio of 1.12. We recorded an average newborn weight of 3209.4 ± 586.6g. Applying the trophic characteristics of “normal” newborns [20] to our study population, we identified 80 (70.2%) “normal” newborns with a mean weight of 3133g ± 347.13 (11.4%) LBW newborns with a mean weight of 2300g ± 221 and 21 (18.4%) macrosomes with a mean weight of 4064g ± 337. We also recorded a total of 5 perinatal deaths (a perinatal mortality of 3.9%).

**Figure 1:**
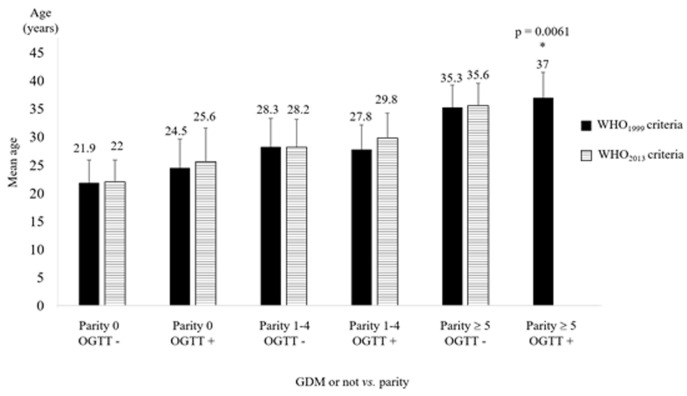
age and parity variation regarding the positivity or not to OGTT (according WHO 1999 and WHO 2013 criteria). Results are mean ± SD

**Identification of factors associated with GDM:** in our study, the ideal gestational age (between 24 and 28 weeks) for performing an OGTT was not associated with the risk of developing GDM (p= 0.8692), although the highest proportion of GDM (11.6%) was found in the group of pregnant women recruited between 24 and 28 weeks. After 28 weeks, we found 9.7% of GDM, and only 8.7% were found in pregnant women less than 24 weeks. The bivariate analyses for identifying factors associated are compiled in Table 4. Furthermore, it is interesting to note that, separately, age and parity, are not factors associated with the occurrence of GDM in the Gabonese population. But taken together, they are. Indeed, our results show that a high age (more than 35 years), combined with high parity (5 parities and more), “taken together” are factors associated with GDM (applying WHO 1999 criteria only) in the Gabonese urban population. The Kruskal-Wallis non-parametric test was applied and showed a relationship between these values (p = 0.0061) (Figure 1).

**Table 4:** the bivariate analyses for identifying associated factors of developing GDM

	Frequency (%)	p vs WHO 1999 criteria	p vs WHO 2013 criteria
**Personal history of GDM**			
Yes	0.8	< 0.0001	0.7582
**Mother's weight at recruitment (Kg)**			
> 77.3		0.0498	0.0905
**Family history of T2D**			
Yes	19.6	0.5579	0.0343
**Mother's age at recruitment (years)**			
(35-and more)	12.7	0.6334	0.9307
**Mother's BMI prior the study (Kg/m^2^)**			
> 25	33.5	0.4652	0.9999
**Mother's BMI at recruitment (Kg/m^2^)**			
> 25	60.4	0.0246	0.3956
**Parity**			
Multiparous (≥ 5)	9.4	0.8203	0.0768

BMI: body mass index; GDM: gestational diabetes mellitus; T2D: type 2 diabetes; Chi2 or fisher's exact test for expected values below 5

**Figure 2:**
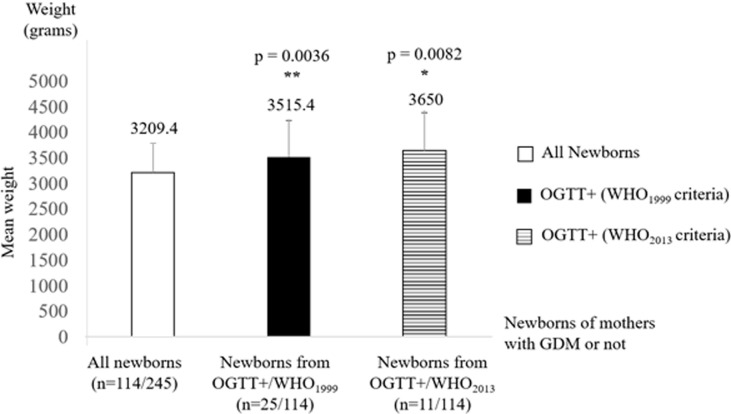
mean newborn weights depending on the mother's health status (± OGTT by applying WHO 1999 or WHO 2013 criteria). Results are means ± SD

**Identification of obstetrical and neonatal outcomes of GDM:** in our study, fetal macrosomia was, as expected, a neonatal outcome of GDM whatever the diagnostic criteria applied (3515.4 ± 722g; p= 0.0036 for WHO 1999 criteria and 3650 ± 729.7g; p= 0.0082 for WHO 2013 criteria), using the student T-test for unmatched series (Figure 2). With regard to the obstetrical outcomes of GDM, C-section was the only maternal complication found, and we only found a relationship by applying the WHO 1999 criteria (p= 0.0334).

## Discussion

Our first challenge was to set the criteria and threshold for the diagnosis of GDM to be applied. We have opted to make a comparative use with our findings, taking into consideration the threshold adopted by the WHO in 1999 and 2013. Prevalence rates of 10.2% and 4.5% respectively with the WHO 1999 and WHO 2013 criteria were found. These findings illustrate a serious problem to diagnose this disease as the difference between the two prevalence rates is considerable. Our results contradict the trend highlighted by several studies at the world level that have found higher prevalences of GDM using WHO 2013 compared to WHO 1999 criteria [19, 21]. In Cameroon, some researchers reported a prevalence of GDM of 6.3% based on the ADA 2010 criteria (T2h ≥ 8.5 mmol/L) [19]. The authors had specified that this prevalence would only have been 3.2% if they had taken into account the WHO 1999 criteria (T2h ≥ 7.8 mmol/L) [20]. Although few data are available, the consensual prevalence of GDM in sub-Saharan Africa is around 9% [9]. Therefore, if we consider the WHO 1999 criteria, the prevalence found in this study (10.2%) becomes alarming not only of sub-Saharan Africa countries but also in other countries with large Caucasian populations such as Denmark (2%), United Kingdom (3%) or Germany (less than 1%) [22]. Nevertheless, concerning the high prevalence of GDM found in other sub-Saharan Africa countries, it is interesting to reveal that, similarly to our study, researches were conducted in urban or suburban areas, especially with at-risk populations. This was the case in Mozambique (11%) and in Nigeria (13.9%) [7, 9]. These results seem to confirm the major impact of the epidemiological transition on the emergence of metabolic diseases in sub-Saharan African urban areas [1]. Altogether, the variation in prevalence rates of GDM worldwide would be due, at least in part, to methodological variations in the diagnosis of this disease since there is no consensus on a universal method of GDM screening. Indeed, this question is still in debate. O'Sullivan *et al*. were first provided evidence that screening, diagnosis and treatment of hyperglycemia during pregnancy in women who did not previously have diabetes improved pregnancy outcomes [23].

However, their diagnostic criteria were later challenged because they were not based on short-term fetal-maternal morbidity, among other things [24]. With the contribution of the Hyperglycemia and Adverse Pregnancy Outcome (HAPO) study, a major step was taken in the screening criteria and the outcome of this disease [25]. Subsequently, the International Association of Diabetes and Pregnancy Study Group (IADPSG) was given the challenge of defining new thresholds for the diagnosis of GDM [17]. The IADPSG validated parameters were subsequently adopted by the WHO and the American diabetes association. Despite these consensual efforts regarding GDM screening, controversy remains and the criteria are still being debated today. In 2015, the National Institute for Health and Care Excellence (NICE) recommended for GDM's screening, new threshold that are different and lower than the IADPSG and the WHO values [26]. For NICE 2015, a 2-step-OGTT with 75g of glucose (T0 and T2h) are sufficient for GDM's screening and the 2h threshold was the same as the WHO 1999, T2h ≥ 7.8 mmol/L [26]. It should be pointed out that, based on the current state of health care provided to pregnant women in Libreville, GDM is not systematically diagnosed and even less treated. During our study, several pregnant women diagnosed with GDM were not sufficiently informed, with the corollary that the recommendations we had made, namely to go to the department of endocrinology at the Libreville University Hospital Centre for appropriate treatment of GDM by an endocrinologist, were not taken into consideration by them [24]. It could explain the explosive prevalence of T2D in the Gabonese population, which has risen from 2.9% to 9.08% in 10 years [27]. Although the impact of insulin therapy in situations of hyperglycemia during pregnancy would depend mainly on the severity of maternal hyperglycemia [24], which is very effective in severe hyperglycemia and very little, if any, in women with less severe hyperglycemia such as GDM [24]. A good alternative would be to implement as recommended, a physical activity compatible with pregnancy [11, 24], in addition to dietary and nutritional modifications to limit weight gain during pregnancy

As for classic factors associated with GDM [10, 11, 28], our study shows that only over-weight and/or obesity at the time of screening, and not before the current pregnancy, personal history of GDM, and family history of T2D were associated factors. Surprisingly, maternal age was not correlated with GDM in our study and becomes an associated factor in our population only if it is combined with high parity (≥ 5, p= 0.0061). This result is a new finding that will now have to be taken into account in the follow-up of great multiparous women in Libreville. Regarding the obstetrical outcomes, C-section was the main obstetrical complication found in our study (p= 0.0334, WHO1999 criteria). In the target group of pregnant women diagnosed with GDM, we found 6 out of 25 C-sections or 24% of cases. In the control group of women without GDM, we counted 4 out of 75 C-sections, representing 5.33% of cases. As expected, we confirmed macrosomia as a fetal outcome of GDM, regardless of the criteria used. These results confirm the adverse impact of this disease on the health of the fetus in the short-term. Indeed, based on the work of Mengue C [20], and taking into consideration our results, we have highlighted a significant difference between the weights of newborns of GDM´s women compared to women without GDM, whatever the WHO criteria considered. This link was stronger when considering the WHO 1999 criteria (p = 0.0036), i.e. a T2h ≥ 7.8 mmol/L and a mean birth weight of 3515g. On the other hand, when considering the WHO 2013 criteria i.e. a T2h ≥ 8.5 mmol/L, the mean birth weight for macrosomia was 3650g, but the link highlighted with the GDM was lower (p= 0.0082).

### Limitations of the study

**In diagnosis:** first, it was very difficult to choose the right criteria and then, the right threshold for the GDM diagnosis. Secondly, pregnant women were rarely fasting on the day of enrollment. Therefore, we had to exclude the T0 time of the OGTT from the exploitation of the results. Thirdly, the prevalences found were very different depending on the criteria used, the risk of not diagnosing possible cases is therefore too great.

In pregnant women follow-up: this has represented the biggest issue in this study. It especially concerned the management of women identified with GDM. Also, the oriented management instructions issued by us were not correctly forwarded on by the midwives at the recruitment sites. In the end, only 114 newborn anthropometric data were collected out of 245 women investigated. Therefore, the results relating to the perinatal outcomes of GDM in Libreville should be considered preliminary but are worth noting. Finally, as recommended by FIGO, we had hoped to perform control OGTT at 6 weeks postpartum [11]. Unfortunately, this control test could only be conducted in one of them.

## Conclusion

Our study on GDM in Libreville (Gabon) highlights, despite the small sample size, the restricted area of investigation and the observational nature of the data presented, that the prevalence of GDM in Libreville is high, taking into account the WHO 1999 criteria compare to the WHO 2013 criteria. This discordance is also found with the identified associated factors. Further studies are needed to better appreciate GDM in Gabon. Meanwhile, we recommend: i) to remain vigilant in the choice of diagnostic criteria by choosing the lowest possible threshold (WHO 1999 criteria, i.e. 75 g OGTT, T2H ≥ 7.8 mmol/L) in order to better manage the highest number of women who may not be diagnosed with GDM applying higher threshold; ii) to implement a targeted screening in all pregnant women over 35 years of age, with a weight greater than 77kg at 24 weeks and in all multiparous women.

### What is known about this topic

- *Black people are more prone to develop gestational diabetes mellitus;*
- *Worldwide, C-section and fetal macrosomia are the main outcomes of this condition;*
- *The prevalence of GDM in Africa varies from 7-12%*.

### What this study adds

- *The prevalence of GDM in Gabon is 10.2% according WHO 1999 and 4.5% according WHO 2013 criteria;*
- *Concerning GDM's associated factors, maternal age becomes one, only combined with high parity*.
